# Offloading treatment is linked to activation of proinflammatory cytokines and start of bone repair and remodeling in Charcot arthropathy patients

**DOI:** 10.1186/s13047-015-0129-y

**Published:** 2015-12-10

**Authors:** Agnetha Folestad, Martin Ålund, Susanne Asteberg, Jesper Fowelin, Ylva Aurell, Jan Göthlin, Jean Cassuto

**Affiliations:** Department of Orthopaedics, CapioLundby Hospital, Göteborg, Sweden; Department of Orthopaedics, Sahlgrenska University Hospital, Mölndal, Sweden; Diabetes Care Unit, Department of Medicine, Frölunda Specialist Hospital, Västra Frölunda, Göteborg, Sweden; Department of Radiology, Sahlgrenska University Hospital, Mölndal, Sweden; Orthopaedic Research Unit, Sahlgrenska University Hospital, Staben, Hus U1, 431 80 Mölndal, Sweden, Göteborg University, Göteborg, Sweden

**Keywords:** Charcot arthropathy, Charcot foot, Bone healing, Fracture, Offloading, Diabetes, Neuropathy, Proinflammatory cytokines, IL-6, IL-8, IL-1beta, TNF-alpha, IL-1 receptor antibody

## Abstract

**Background:**

Proinflammatory cytokines are an integral part of the osteolytic activity of Charcot arthropathy but are also central to normal bone healing. As there are no previous longitudinal studies investigating their role during the recovery phase of Charcot, we set out to monitor systemic levels of proinflammatory cytokines from Charcot presentation until a clinically and radiographically documented chronic state has been reached.

**Methods:**

Twenty-eight consecutive Charcot patients were monitored during 2 years by repeated foot radiographs, MRI and plasma levels of interleukin [IL]-6, IL-8, IL-1β, Tumor Necrosis Factor [TNF]-α, and IL-1 receptor antibody (IL-1RA). Charcot patients were treated with total contact cast (TCC) on the first day of inclusion. Neuropathic diabetic controls (*n* = 20) and Healthy subjects (*n* = 20) served as reference.

**Results:**

Plasma IL-6, IL-8, IL-1β and TNF-α in the acute and chronic phase of Charcot were below or at the level of diabetic controls and healthy, whereas IL-1RA/IL-1β ratio was continuously higher in Charcot patients. IL-6, TNF-α and IL-1RA began to increase one week after offloading to reach a peak after 4 months before gradually receding.

**Conclusions:**

A sustained increase of IL-6 and TNF-α starting shortly after offloading and paralleled by accelerated bone healing on radiographs, suggest that offloading, by activating the inflammatory stage, has a key role to play in the onset of coupled bone remodeling. High IL-1RA/IL-1β ratio in Charcot patients at presentation supports a counter-balancing anti-inflammatory role for IL-1RA in the acute phase whereas a high ratio after two years, possibly due to renewed weight-bearing on a deformed foot, signal need for continued anti-inflammatory activity and contradicts a “cold” biological state in the chronic phase.

## Background

**Fig. 1:**
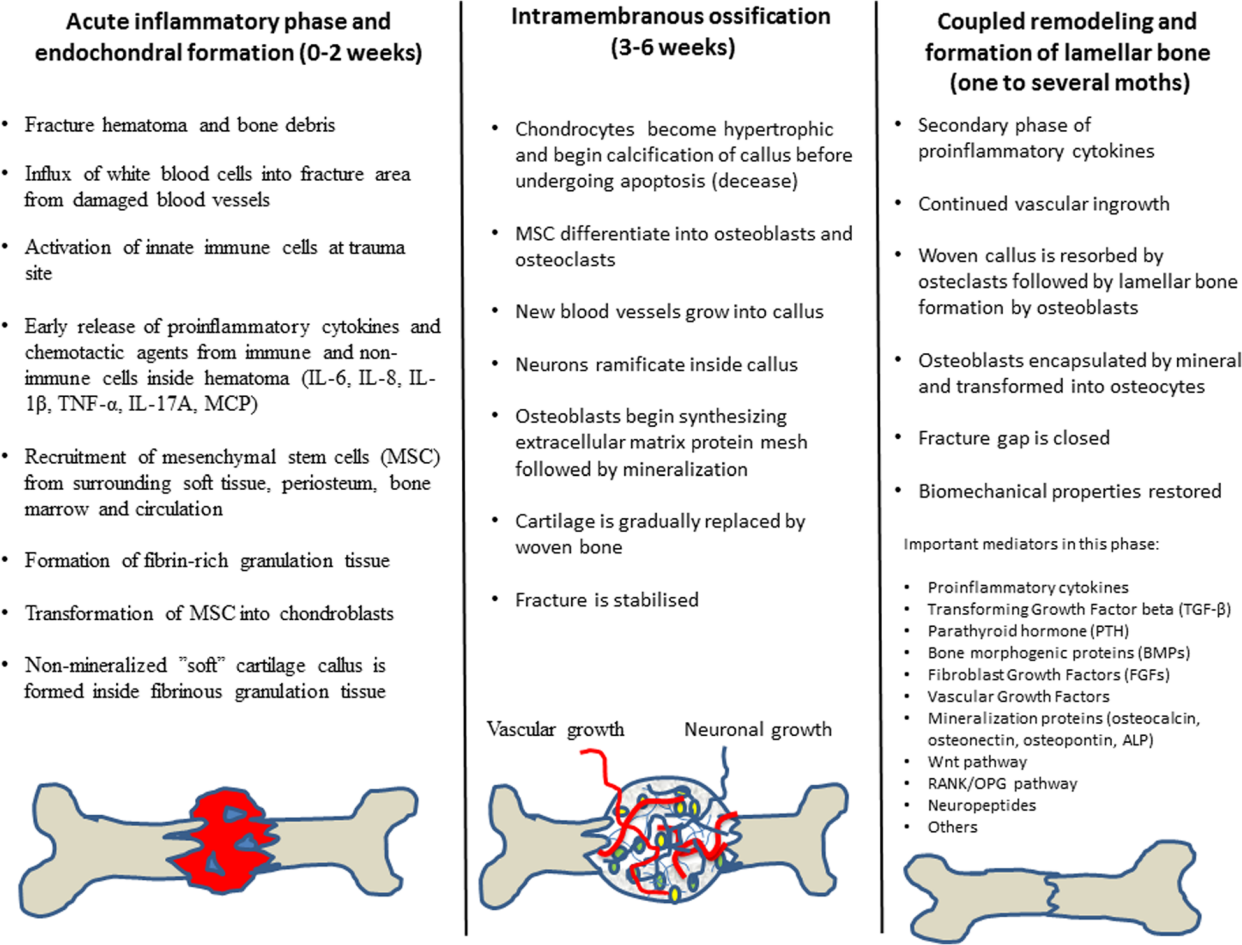
Schematic overview of the main phases of bone fracture repair in humans

The Charcot foot is often characterized by local inflammation, diastases, joint dislocations, and fractures. A great number of experimental and clinical studies have shown that most fractures heal by overlapping phases characterized by inflammation, bone regeneration and remodeling and that these phases normally follow a preset schedule with a rather controlled set of events when fracture healing is allowed to proceed undisturbed (Fig. 1) [1, 2]. In the primary phase of fracture healing, proinflammatory cytokines, such as IL-6, IL-8, IL-1β, IL-17 and TNF-α, are expressed and released within the first hours or days after tissue injury. This initial cytokine boost from the damaged bone and soft tissue is transitory in nature and only lasts a few days but yet is responsible for a number of key events such as removal of necrotic tissues and recruitment of neutrophils and mesenchymal stem cells [1, 3]. Stem cells are stimulated to undergo differentiation into chondrocytes leading to formation of cartilaginous callus that stabilizes the fracture and, when absent or disturbed, will have detrimental effects on mesenchymal cell maturation and callus formation [3]. An intermediate phase of low activity by proinflammatory cytokines follows and is characterized by cartilaginous callus being calcified and replaced by woven bone. A secondary or late phase of expression and release of proinflammatory cytokines will ensue and stimulate the transformation of stem cells into osteoblasts and osteoclasts allowing for the coupled remodeling phase characterized by woven bone of the callus being replaced with lamellar [1, 3].

We currently know little of the mechanisms triggering the pathological changes characteristic of Charcot arthropathy although trauma to the foot and the ensuing inflammatory reaction has been proposed as an important driving force [4]. Although the acute Charcot foot presents itself with the classical features of a local inflammatory reaction, i.e. a red, hot and swollen foot [5], the pathogenic role of inflammation can be multifaceted as it may both play a primary role in triggering the onset of the destructive processes leading to the anatomical changes of foot, or be secondary to these changes [6]. Inflammation, being a general response by the body to all types of trauma, serves as an important part of the repair and defense mechanisms, before subsiding when the cause of inflammation has been removed or the healing process has been allowed to proceed uninterrupted. A critical factor for the Charcot patient in this context is their loss of pain as protective sensory input and thus inability to identify the severity of trauma which will often delay the onset of medical treatment by weeks or months. This, in turn, will lead to continued weight-bearing on the diseased foot with motion at fracture site causing delayed bone union which has led clinicians globally to adopt a treatment strategy of prolonged non-weight-bearing to create better healing conditions [7, 8]. As proinflammatory cytokines have been shown to play a pivotal role in bone repair and there are no previous longitudinal studies investigating their role in Charcot patients we set out to monitor systemic levels from presentation until a clinically and radiographically documented cold/chronic state has been reached.

## Methods

This observational prospective study was approved 2007 by the Review Board of Västra Götalands Regionen (EPN D-number 499–07) with additional approval 2010 (T 762–10 ad 499–07). All the subjects provided informed consent before participating in the study. The study complies with the STROBE-statement for observational studies [9].

### Patient selection and treatment

Twenty-eight Thirty consecutive ambulatory men and women admitted to Sahlgrenska University Hospital/Mölndal with clinical signs of unilateral acute Charcot’s foot were included in the study during 2009–2013. Two patients deceased shortly after inclusion and were excluded. 1 patient interrupted participation after 18 months after being informed that radiographs showed finalized bone healing, but did not object to data being processed in the study. Diagnosis of Charcot foot was based on medical history, clinical examination and radiological findings including the following criteria: (1) Type 1 or 2 diabetes with duration of ≥ 1 year (2) peripheral bilateral neuropathy as defined below and (3) clinical signs of active Charcot arthropathy with hot/reddened/swollen foot and skin temperature in the affected foot ≥ 2 °C higher than the contralateral foot. Off-loading treatment of the diseased foot by a non-weight bearing total contact cast (TCC) was introduced in all Charcot patients within 1 day of inclusion to the study. The TCC was repeatedly replaced as required by changes in foot volume due to increased or attenuated swelling. The non-weight-bearing protocol was aided by crutches or wheel-chair and was continued until the difference in skin temperature between the feet was ≤ 1 °C and no signs of redness and swelling were present for the past 30 days. TCC was then replaced by orthosis for partial weight-bearing. Evaluation of skin temperature and toe pressure continued until 2 years postinclusion. When full weight-bearing was allowed, the patient received prescribed accommodative shoes. Bilateral foot radiograhs and magnetic resonance imaging (MRI) at inclusion and repeatedly during the follow-up period were used to monitor fractures, dislocations and soft tissue-or bone marrow edema. Exclusion criteria were plantar ulcerations, documented history of trauma or surgery involving bone tissue during the past year prior to inclusion, current immunosuppressive therapy (steroids, cancer treatment) or medication known to affect bone metabolism (e.g. bisphosphonates, denosumab). Considering the detrimental effects on bone healing attributed to diabetes and peripheral neuropathy (see Discussion), a control group of 20 ambulatory diabetes type 1 or 2 patients with documented peripheral neuropathy was recruited at Frölunda Specialist Hospital thus allowing us to distinguish changes occurring as a result of Charcot arthropathy and its treatment. A group of healthy subjects was recruited at Sahlgrenska University Hospital/Mölndal and served as a reference to the neuropathic diabetic control group as we were previously able to show important differences in bone regulating cytokines between these groups [10]. Both control groups lacked history of joint/bone disease or bone trauma/surgery 1 year prior to inclusion and received no osteoporosis medication.

### Skin sensitivity, skin temperature and toe pressure

Peripheral neuropathy was assessed bilaterally by measuring foot skin sensitivity in Charcot and diabetic control patients using the Semmes-Weinstein monofilament test. The monofilament (10 g) was pressed against 4 different locations on the foot and the patient’s ability or inability to feel the sensation upon buckling of the monofilament was registered. Neuropathy was present if 3 or more sites were insensate to the monofilament. Skin temperature was measured bilaterally on the dorsal foot 5 cm distal of the ankle and 2 cm proximal of the mid toe. Toe pressure was measured bilaterally using a specially designed cuff [11]. Measurements of skin temperature and toe pressure were performed at inclusion, after 1 week, 2, 4, 6, 8, 12, 18 and 24 months.

### Blood samples

Peripheral blood samples were collected for routine analysis (Hemoglobin, serum creatinine, CRP, SR and HbA1c). Venous blood for biomarker analysis was sampled in pre-chilled EDTA tubes which were immediately centrifuged for 10 min at 4 °C and plasma was stored at −85 °C until analysis. Blood was collected on the day of inclusion, after 1 week, 2, 4, 6, 8, 12, 18 and 24 months. Before analysis of IL-6, IL-8, TNF-α, IL-1β and IL-1RA, samples were thawed on ice and mounted on assay plates. The IL-1RA/IL-1β ratio was calculated from IL-1RA and IL-1β data in individual patients obtained by analysis of the same batch of patient plasma at each specific time point. Blood from diabetes control patients and healthy subjects was sampled on one occasion as we did not expect clinical and laboratory variables to undergo significant changes in these groups during the short duration of the study. Frequent routine laboratory monitoring in the diabetes control group enabled us to compare laboratory data at inclusion and 2 years later confirming that no significant laboratory/clinical changes had occurred in this group during the course of the study (Table 1). Biomarker data from diabetic controls and healthy subjects were thus used as reference for the Charcot group both at inclusion and at 2 years postinclusion.

**Table 1:** Clinical and demographic data in Charcot patients, diabetic controls and healthy subjects

	Charcot arthropathy inclusion (*n* = 28)	Charcot arthropathy end of study (*n =* 27)	Diabetic controls inclusion (*n* = 20)	Diabetic controls +2Y (*n* = 20)	Healthy subjects (*n* = 20)
Age (median- range, years)	61 (42–87)	63 (42–87)	55 (20–94)	57 (20–94)	58 (24–78)
Gender (n)					
Females	1012	101011	8	8	9
Males	16	16	12	12	11
Diabetes type 1/2 (n)	11/1517	11/1516	6/14	6/14	-
Diabetes duration (years)	25 ± 4	-	14 ± 6	-	-
Debut foot symptoms (weeks, median-range)	8.4 (1–24)	-	-	-	-
Systolic blood pressure (mmHg)	149 ± 6	148 ± 6	127 ± 4	-	138 ± 4
Diastolic blood pressure (mmHg)	80 ± 3	80 ± 3	76 ± 2	-	81 ± 3
Arterial toe pressure (mmHg)					
Charcot foot	114112 ± 11	122 ± 8	115 ± 4	-	-
Contralateral foot	129128 ± 8	119 ± 7	114 ± 5	-	-
Skin temperature (°C)					
Charcot foot	31.331.6 ± 0.8^a^	29.7 ± 0.7	30,0 ± 0,4	-	-
Contralateral foot	28.8 ± 0.9	29.3 ± 0.7	30,2 ± 0,4	-	-
Pain at rest (n)	4	0	0	-	-
Pain at weight bearing (n)	17	0	0	-	-
Foot distorsion (n)	4	4	0	-	0
Total contact cast (months)	-	7.7 ± 1.0	-	-	-
Charcot pattern I/II/III/IV/V (n)	2/16/57/0/3	-	-	-	-
Nephropathy/Cardiac disease (n)	7/4	7/4	2/1	2/1	0/0
Hb (g/L)	127 ± 3	129 ± 3	136 ± 2	139 ± 3	143 ± 5
S-CRP (mg/L)	10.611.2 ± 3^b,c^	10.09.8 ± 2^d,e^	3.6 ± 0,8	3.8 ± 0,8	2.7 ± 0,7
SR (mm)	28.831.1 ± 6^f,g^	23 ± 5	11 ± 2	10 ± 2	6 ± 4
S-Kreatinin (μmol/L)	112116 ± 19	101 ± 12	83 ± 8	89 ± 7	78 ± 5
HbA1c (mmol/mol)	60 ± 3	64 ± 2	58 ± 3	56 ± 2	-
HbA1c (%)	7.8 ± 0.3	7.4 ± 0.3	7.5 ± 0.2	7.3 ± 0.2	-

Mean ± SEM when not given differently. ^a^ *p* = 0.0100.0100.007 Charcot foot versus contralateral foot at inclusion. ^b^ *p* = 0.028 for Charcot at inclusion versus diabetic controls at inclusion. ^c^ *p* = 0.015 for Charcot at inclusion versus healthy. ^d^ *p* = 0.0380.030 Charcot end of study vs diabetic controls +2y. ^e^ *p* = 0.022 Charcot end of study vs healthy. ^f^ *p* =< 0.001 Charcot at inclusion versus diabetic controls at inclusion. ^g^ *p* < 0.001 for Charcot at inclusion versus healthy. All other differences are not significant. Charcot pattern I-V on radiographs as described by Sanders and Frykberg (see ref: Papanas and Maltezos, [13])

### Determination of plasma biomarkers by ECL technology

The Sector Imager 2400 assay platform from MesoScaleDiagnostics (MSD, Gaithersburg, USA) was used for analysis of plasma biomarkers (for details see www.mesoscale.com). This high sensitivity electrogenerated chemiluminescence technique (ECL) has a low limit of detection and a wide dynamic range as was described in more detail in a previous study [12]. Matched pairs of antibodies, i.e. capture antibody (CA) and biotinylated detection antibody (DA), were used. Standard curves were created using human recombinant proteins (hRP). Plasma was mounted on uncoated plates from MSD (L15XA). IL-8 CA: monoclonal mouse IgG1κ (PeproTech cat no. 500-M08), IL-8 DA and hRP (PeproTech cat no. 900-K18). IL-6 CA: monoclonal mouse IgG1 (Biotechne R&D Systems cat no. MAB2062), IL-6 DA: affinity purified polyclonal goat IgG (Biotechne R&D Systems cat no. BAF206). TNF-α CA/DA/hRP: PeproTech cat no. 900-K25. IL-1β CA: monoclonal mouse IgG1 (Biotechne R&D Systems cat no. MAB601), IL-1β DA: affinity purified polyclonal goat IgG (Biotechne R&D Systems cat no. BAF201), IL-1β hRP (Prospec cat no. cyt-208). IL-1RA CA/DA/hRP: PeproTech cat no. 900-K474. Antibodies were optimized by checkerboard titrations and subsequent control of standard curves. Plasma from Charcot, diabetic controls and healthy subjects were mounted on the same plate to minimize inter-group variability. Inter-assay variations were <5 %.

### Radiography

Radiographs of both feet were performed in supine position with dorso-plantar, oblique and lateral projections as well as weight-bearing in frontal and lateral projections. Examinations followed a preset schedule starting within a week after inclusion and subsequently 6, 12, 18 and 24 months postinclusion. MRI was performed on a 1.5 T Siemens Magnetom Symphony with supine patient and feet first into the gantry. All examinations were performed with a head-neck surface coil and feet in flexed position. Each foot was examined with 4 sequences without intravenous contrast medium: T1, T2, T2 3d and STIR sequences in sagittal, transverse and coronary positions. Intravenous contrast was followed by T1-sequences in transverse and sagittal projections.

### Charcot classification

For anatomical classification of Charcot we used the system described by Sanders and Frykberg with five different patterns depending on the areas of the foot affected [13]: Pattern I: metatarsophalangeal and interphalangeal joints; Pattern II: tarso-metatarsal joints; Pattern III: naviculocuneiform, talonavicular and calcaneocuboid joints; pattern IV: ankle and subtalar joints; Pattern V: calcaneus (Table 1). A modified Eichenholz staging based on plain X-rays as described by Sella and Barrette [14], was used for disease characteristics and comprises 5 stages: Stage 0 (warm, reddened, swollen foot and normal radiographs), stage 1 (clinical findings and radiographic cysts, erosions, localized osteopenia and occasionally diastases), stage 2 (joint subluxation), stage 3 (dislocation and arch collapse), stage 4 (healed stage of bony process) (Table 1). Inflammation in the soft-tissue and bony structures of the foot (edema), were identified by MRI and summarized in Table 2.

**Table 2:** Staging of radiographic changes in the Charcot arthropathy foot as described by Sella and Barrette [14] based on weight-bearing radiographic examinations of the diseased foot and inflammation/bone edema on MRI

Radiographs	Inclusion	1 W	6 M	12 M	18 M	24 M
Stage 0	4	3	0	0	0	0
Stage 1	3	3	2	0	0	0
Stage 2	911	911	911	68	45	0
Stage 3	10	10	10	5	5	0
Stage 4	0	1	5	15	17	2527
MRI	Inclusion	1 W	6 M	12 M	18 M	24 M
Soft tissue edema	2223	2223	1819	14	7	0
Bone marrow edema	18	18	15	13	5	0

Numbers in table are number of patients

### Statistical methods

As the assay platform used for biomarker analysis in the current study is significantly more sensitive than that of colorimetric ELISAs, we chose to base our power analysis on the standard deviations (SD) obtained from pre-study measurements of individual cytokines. A mean of the SD of all the individual cytokines of the study was used as a basis for calculation of sample size. ANOVA test of 3 groups with a minimum detectable difference in means (=20), mean SD (=32), power (=0.80) and alpha (=0.05) required 51 patients.

The tested variables (biomarkers) were measured at the continuous level over time and a comparison was made between the 3 independent study groups for each individual biomarker at inclusion and at 2 years postinclusion by means of 1-way repeated measures ANOVA followed by the Holm-Ŝidak test. When applicable (normally distributed data), this model was also used for comparisons between the 3 study groups for blood pressure, Hb, CRP, SR and S-kreatinin (Table 1). This statistical model ensures that the probability of incorrectly rejecting the null hypothesis for any of the pairwise comparisons in the family does not exceed alpha, thereby controlling the familywise error rate (FWE) and setting the alpha value according to the Ŝidak correction of Bonferroni inequality. This stepwise method is generally more powerful than the corresponding single-step procedures. The Holm-Ŝidak test applies an accept/reject criterion on a sorted set of null hypothesis, starting from the lower *p*-value and going up to the acceptance of null hypothesis. The Ŝidak formula is: Ŝidak *corrected alpha* = 1 - (1 - α)^1/*k*^ where *k* (=3 in our study) is the number of comparisons performed for each individual biomarker. The Ŝidak procedure has slightly more power than the Bonferroni procedure when alpha = .05. A multiplicative model was assumed more accurate for the current data and therefore the logarithms of measurements have been used for the comparisons. Comparisons between 2 datasets of individual biomarkers within the Charcot group (e.g. inclusion vs. 4 months) and comparison between groups (Charcot and diabetic controls) of a variable that failed normal distribution (HbA1c), were done by the non-parametric Wilcoxon rank sum test (Table 1).

## Results

Clinical and demographic data are presented in Table 1. Radiographic data are presented in Table 2. Tables 1 and 2 have in part been presented in a previous study [12]. One patient had no radiographic data after 18 months into the study as the patient chose to interrupt participation.

### IL-6

**Fig. 2:**
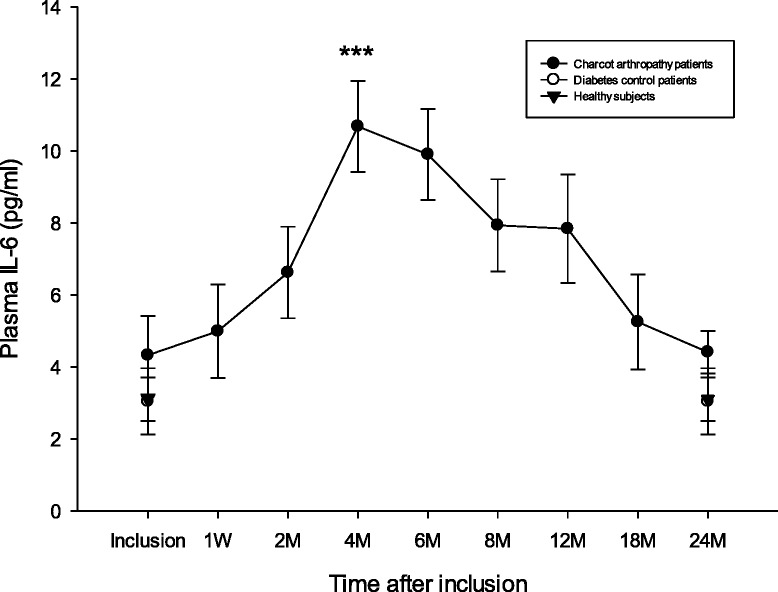
Plasma IL-6 (pg/ml) in Charcot patients (*n* = 28), diabetes control patients (*n* = 20) and healthy donors (*n* = 20). IL-6 was not significantly different between the 3 groups at inclusion (*p* = 0.64) or at 2 years (*p* = 0.22). ***P < 0.001 for Charcot at 4 months versus Charcot at inclusion. Charcot at 2 years was not different from Charcot at inclusion (*p* = 0.19). Mean ± SEM

Plasma IL-6 (pg/ml) was not significantly different between Charcot patients, diabetic controls and Healthy at inclusion (*p* = 0.64) or at 2 years (*p* = 0.22). Plasma IL-6 in Charcot patients was significantly elevated at 4 months versus inclusion (*p* < 0.001) but did not differ significantly between inclusion and 2 years postinclusion (*p* = 0.19) (Fig. 2).

### IL-8

**Fig. 3:**
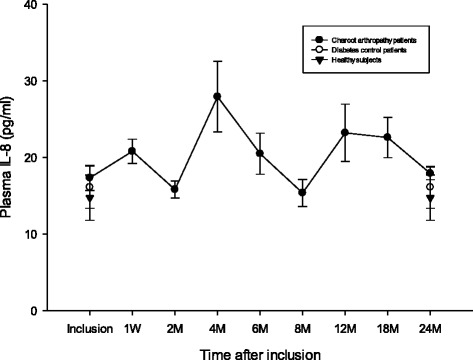
Plasma IL-8 (pg/ml) in Charcot patients (*n* = 28), diabetes control patients (*n* = 20) and healthy donors (*n* = 20). IL-8 was not significantly different between the 3 groups at inclusion (*p* = 0.83) or at 2 years (*p* = 0.45). IL-8 in Charcot patients at inclusion did not differ from Charcot at 4 months (*p* = 0.91) or at 2 years (*p* = 0.35). Mean ± SEM

Plasma IL-8 (pg/ml) was not significantly different between Charcot patients, Diabetes control patients and Healthy subjects at inclusion into the study (*p* = 0.83) or at 2 years postinclusion (*p* = 0.45). Plasma IL-8 in Charcot patients had not changed significantly at 4 months (*p* = 0.91) or at 2 years (*p* = 0.35) versus inclusion value (Fig. 3).

### TNF-α

**Fig. 4:**
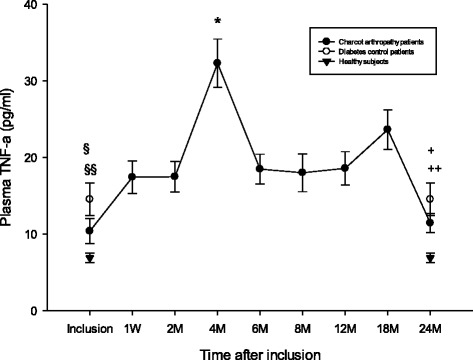
Plasma TNF-α (pg/ml) in Charcot patients (*n* = 28), diabetes control patients (*n* = 20) and healthy donors (*n* = 20). §*P* = 0.005 for diabetic controls versus healthy, §§*P* = 0.005 for diabetic controls versus Charcot. Difference between Charcot at inclusion and diabetic controls was not significant (*p* = 0.64). *P = 0.02 for Charcot at 4 months versus Charcot at inclusion. +*P* = 0.001 for diabetic controls versus healthy, ++P = 0.015 for diabetic controls versus Charcot. Difference between Charcot and healthy at 2 years was not significant (*p* = 0.53). Charcot at 2 years was not different from Charcot at inclusion (*p* = 0.44). Mean ± SEM

Plasma TNF-α (pg/ml) in diabetic controls was significantly higher at inclusion versus healthy (*p* = 0.005) and versus Charcot (*p* = 0.005) but did not show significance between Charcot and healthy (*p* = 0.64) (Fig. 4). At 2 years postinclusion, plasma TNF-α was significantly higher in diabetic controls versus Charcot (*p* = 0.001) and versus healthy (*p* = 0.015) whereas difference between Charcot and healthy was not significant (*p* = 0.53). TNF-α in Charcot patients was significantly higher at 4 months versus inclusion (*p* = 0.02) but not at 2 years versus inclusion (*p* = 0.44).

### IL-1β

**Fig. 5:**
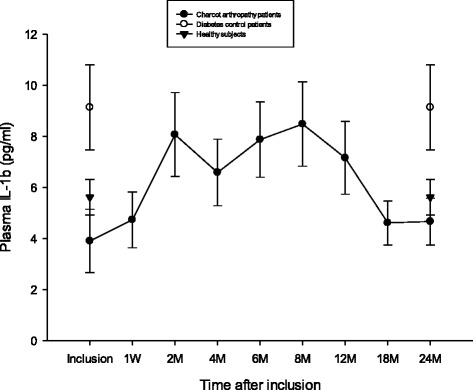
Plasma levels of IL-1β (pg/ml) in Charcot patients (*n* = 28), diabetes control patients (*n* = 20) and healthy donors (*n* = 20). §P=0.029 for diabetic controls versus Charcot. Differences were not significant between diabetic controls and healthy (*p*=0.16) or between Charcot and healthy (*p* = 0.29). +P=0.027 for diabetic controls versus Charcot. Differences were not significant for diabetic controls versus healthy (*p*=0.016) or between Charcot and healthy (*p* = 0.28). Charcot at inclusion was not different from Charcot at 4 months (*p* = 0.07) and or at 2 years (*p* = 0.26). Mean ± SEM

Plasma IL-1β at inclusion was significantly higher for diabetic controls vs. Charcot (*p* = 0.029) but not vs. healthy (*p* = 0.16) or between Charcot and healthy (*p* = 0.29) (Fig. 5). At 2 years postinclusion, IL-1β was significantly higher in diabetic controls versus Charcot (*p* = 0.027) but not versus healthy (*p* = 0.16) or between Charcot and healthy (*p* = 0.27). IL-1β in Charcot patients was not significantly elevated at 4 months versus inclusion value (*p* = 0.06) or at 2 years versus inclusion (*p* = 0.26).

### IL-1RA

**Fig. 6:**
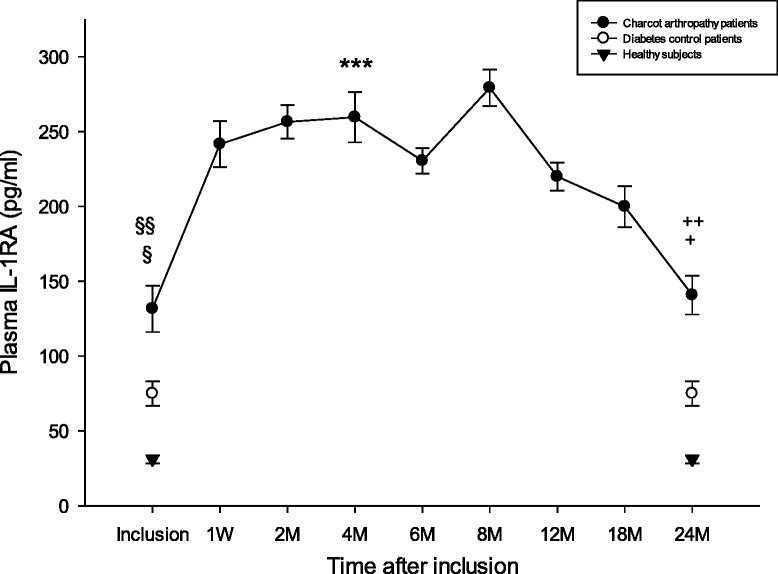
Plasma levels of IL-1RA (pg/ml) in Charcot patients (*n* = 28), diabetes control patients (*n* = 20) and healthy donors (*n* = 20). §*P* = 0.034 for diabetic controls versus healthy, §§*P* = 0.004 for Charcot versus healthy. Difference between Charcot at inclusion and diabetic controls was not significant (0.45). ***P < 0.001 for Charcot at 4 months versus Charcot at inclusion. +*P* = 0.017 for diabetic controls versus healthy, ++*P* < 0.001 for Charcot versus healthy. Difference between Charcot at inclusion and Charcot at 2 years was not significant (*p* = 0.85). Mean ± SEM

Plasma IL-1RA at inclusion was significantly lower for healthy versus diabetic controls (*p* = 0.034) and versus Charcot (*p* = 0.004) but did not differ significantly between Charcot and diabetic controls (*p* = 0.45) (Fig. 6). At 2 years postinclusion, IL-1RA was significantly lower for healthy versus diabetic controls (*p* = 0.017) and versus Charcot (*p* < 0.001) but did not differ significantly between Charcot and diabetic controls (*p* = 0.20). IL-1RA in Charcot patients was significantly higher at 4 months versus inclusion (*p* < 0.001) but not at 2 years vs. inclusion (*p* = 0.85).

### IL-1RA/IL-1β ratio

**Fig. 7:**
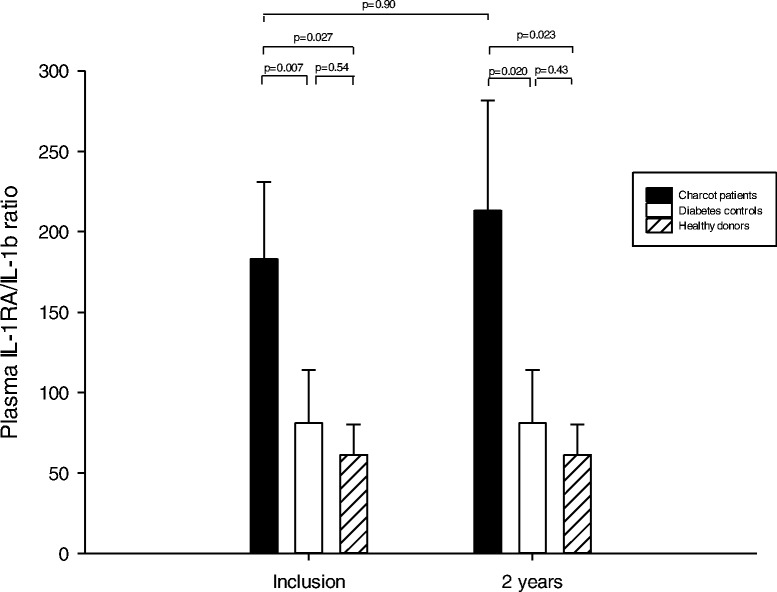
IL-1RA/IL-1β ratio in Charcot patients (*n* = 28), diabetes control patients (*n* = 20) and healthy donors (*n* = 20)

The ratio was significantly higher in Charcot patients at inclusion versus diabetic controls (*p* = 0.007) and versus healthy (*p* = 0.027), but not between diabetic controls and healthy (*p* = 0.54) (Fig. 7). At 2 years postinclusion, the ratio was significantly higher in Charcot patients versus diabetic controls (*p* = 0.020) and versus healthy (*p* = 0.023), but not between Charcot and healthy (*p* = 0.43). Ratio in Charcot patients did not differ between inclusion and 2 years (*p* = 0.90).

## Discussion

Although radiographs showed significant bone pathology in a majority of Charcot patients at presentation (Table 2), we were only able to identify four Charcot patients with visible callus on radiographs despite that 12 weeks had elapsed since debut of symptoms (8 weeks for the Charcot group as a whole). The time for bony callus to appear on radiographs may depend on the location of a fracture but will in most cases be visible within 3 weeks of the initial trauma. It is thus conceivable to assume that bony healing had failed to materialize in most of our Charcot patients with little proof of progress beyond the endochondral phase. Several factors could account for this lack of progress. One is the diabetic disease *per se* which, aside from being associated with higher incidence of fractures than the population as a whole [15, 16], is prone to develop complications in the sequel of a fracture by as much as 87 % and often heal with impaired bone quality [17–19]. Accumulation of highly reactive agents in diabetic patients, such as advanced glycation end products (AGE), reactive oxygen species (ROS) and proinflammatory cytokines (particularly TNF-α), have been linked to these complications. Besides prolonging the survival of osteoclasts and inducing cell death (apoptosis) of chondrocytes resulting in premature destruction of the cartilage callus, these agents also inhibit the differentiation of osteoblasts thereby hampering the subsequent mineralization and remodeling of callus [20]. Another co-morbidity of Charcot with a potential of negatively affecting bone healing on multiple levels is the mandatory neuropathy of the lower limbs [18, 21]. Neuropathic diabetic patients may suffer vitamin D deficiency causing impaired mineralization of the bone, have abnormal blood flow pattern in the lower extremities, higher incidence of atherosclerotic changes in peripheral arteries, and reduced numbers of periostal mechanoreceptors of importance for fracture union [18, 21]. In addition, recent studies have revealed that neuropeptides from autonomic and sensory neurons are able to influence bone formation and fracture healing by direct actions on osteoblastic and osteoclastic cells [21]. It is however the impaired sensitivity in the neuropathic foot which is considered to be of paramount importance for for the development of Charcot arthropathy [22] as the patients, unable to recognize the severity of ongoing pathology, continue to weight-bear on the diseased foot [18, 21], thereby complicating the already complex dynamics of inflammation and fracture healing. A significant and rapidly expanding knowledge base of signaling pathways and the complex cross-talk between these pathways has continuously added to our understanding of the role played by the innate and adaptive immune systems, enzymes and hormones in bone repair and remodeling [3, 23]. This has led to steadily increasing numbers of biological agents targeting specific components of the bone repair cascade with the aim of improving the outcome [24]. Nonetheless, our current understanding of the biological mechanisms regulating the pathology and recovery of Charcot arthropathy is almost entirely circumstantial in nature. In two recent studies to be discussed below, we presented evidence of the important role played by proinflammatory cytokines belonging to the Th17 subset of T helper cells [12] and by mediators of two major bone-regulating pathways [10] for bone healing in Charcot arthropathy patients. In the current study, we extended our investigation to the THh1/Th2 subset of proinflammatory cytokines.

A comparison of diabetic neuropathic control patients and with healthy individuals revealed no significant differences as for IL-6, IL-8 and IL-1β, whereas TNF-α was significantly higher in diabetic controls. Although previous studies have linked diabetes to high systemic levels of proinflammatory cytokines [25–27], therapeutic regulation of hyperglycemia has proven a rather effective measure of normalizing cytokine levels [25, 26] which could suggest that the reports showing systemic proinflammatory cytokines in diabetic patients to range from lower to equal or higher than healthy individuals are linked to the duration and state of the patient diabetic disease at the time of sampling, although differences in assay type and sensitivity could have played in [28]. In two previous studies investigating monocytes from acute Charcot patients, results showed increased levels of proinflammatory immune phenotype [29] and increased numbers of CD-14 positive monocytes with a potential of transforming into osteoclasts [30]. They also showed slightly but significantly elevated serum IL-6, TNF-α and IL-1β in Charcot versus healthy [29] and higher TNF-α but not IL-1β in Charcot versus diabetic controls and healthy [30]. Our results showing all proinflammatory cytokines in Charcot patients at presentation to be below or at the level of diabetic controls are thus in opposition to the previous studies. We cannot offer a plausible explanation to these differences but emphasize that our study had a larger number of patients (66 in our study vs. 27 by Mabilleau and 26 by Uccioli) and used a significantly more sensitive technique to analyze biomarkers having with a dynamic range of 10^6^ as compared to 10^3^ for colorimetric ELISA [31, 32]. It may appear paradoxical that proinflammatory cytokines in Charcot patients at presentation are below or at the level of diabetic controls when considering that the acute Charcot foot presents itself with soft tissue, cartilage and bone pathology and the classical features of a local inflammatory reaction, i.e. a red, hot and swollen foot [5]. A plausible explanation could be that proinflammatory cytokines such as TNF-α and IL-1β, although important for the initiation of tissue inflammation following trauma [1, 3], only last a few days and constitute a small portion of the great basin of mediators (e.g. bradykinin, histamine, prostaglandins, substance P) involved in the local regulation of blood flow (flare and heat reaction) and vascular permeability (swelling) [33] with the ability to entertain the complex neuro-inflammatory cascade until optimal healing conditions are met. Another explanation for the low levels of proinflammatory cytokines at Charcot presentation is the counter-regulatory activation of anti-inflammatory cytokines, such as IL-4, IL-10 and IL-13, in order to down-regulate potentially harmful levels of proinflammatory cytokines [34, 35]. The latter mechanism is supported by the current results showing high levels of IL-1RA, the circulating natural antagonist of the potent proinflammatory cytokine IL-1β. IL-RA acts as a counter-regulatory positive feed-back loop with direct inhibitory effect on IL-1β thereby protecting the bone from excessive osteoclastic activity [36]. However, as we also showed high IL-1RA level in diabetic control patients, it could be inferred that its activity is linked to the diabetic disease *per se* as high inflammasome activity and excessive release of ROS, typical of diabetes [37], have been shown to induce high levels of IL-1RA [38]. Diabetes as the sole explanation is however contradicted by the IL-1RA/IL-1β ratio being significantly higher in Charcot patients due to lower IL-1β being lower than in diabetic controls. A high ratio at presentation and further increase during offloading suggest that IL-1β had reached a harmful level in the diseased foot thus prompting a counter-regulatory inhibition and a shift of the ratio in an anti-inflammatory direction. This inhibition of IL-1β may also explain the contained levels of IL-6 and TNF-α, as most proinflammatory cytokines which are present at high levels in the blood of diabetic patients are IL-1-driven and reduced by blocking its activity [39]. This inhibition appear however to extend beyond the Th1/Th2 subset of proinflammatory cytokines as we were recently able to show a similar containment of IL-17 cytokines belonging to the Th17 subset in Charcot patients exerting full weight-bearing at presentation [12].

Although minor movements in a fracture have been proven beneficial for bone healing, instability and excessive motion has been shown to cause predominantly catabolic activity, suppress ingrowth of new blood vessels, trigger excessive formation of cartilage and inhibit its subsequent replacement with bone thus hindering bone from bridging the fracture gap [3]. Stabilization and offloading of the Charcot foot in order to create a better bio-environment for recovery has therefore emerged as the most important strategy to prevent further deterioration and reverse the pathological processes [5]. Such a mechanism could explain the current results showing that IL-6 and TNF-α had begun to increase shortly after TCC treatment when unfavorable healing conditions caused by continued weight-bearing on the diseased foot had ceased. A similar increase by IL-17 family cytokines shortly after TCC in Charcot patients [12] lend further support to offloading being a critical factor which interrupts the vicious cycle caused by continued weight-bearing and sets the wheels of bone repair in motion. Although it may seem paradoxical that increased activity by inflammatory cytokines is beneficial to the healing process, there is ample evidence to show that a secondary or late phase of proinflammatory mediators is in fact mandatory for bone healing to proceed normally [3, 40]. Proinflammatory cytokines, such as TNF-α, IL-1β, IL-6 and IL-17, have been shown to play a pivotal role in the initiation of bone mineralization and remodeling as they, in addition to inducing osteoclast differentiation, also stimulate angiogenesis and promote osteoblast differentiation and when lacking cause fracture healing to be significantly delayed [1, 3]. The importance of offloading is further emphasized by recent results showing that TCC treatment coincided with a significant increase by dickkopf-1 (dkk-1) and Wnt ligand-1 (Wnt-1) [10], two mediators of the bone anabolic Wnt/β-catenin pathway implicated in bone remodeling [41, 42] and that an inter-regulatory link exists between proinflammatory cytokines and the Wnt system [43]. Our results showing that IL-8 did not change in the aftermath of offloading treatment could suggest that it had outplayed its most important role as chemotactic agent, i.e. to attract leukocytes, in the early post-injury phase. An interesting observation of the study was that although radiographs and MRI showed mineralized bone and no visible edema after 2 years, IL-1RA/IL-1β ratio remained high thus signifying a need for continued anti-inflammatory activity. A plausible reason for such a defensive measure in the chronic phase of Charcot could be that renewed weight-bearing on the Charcot foot, often characterized by bone deformities, will lead to unnatural high pressure on areas of the foot leading to exacerbated inflammation and microdamage [44]. The chronic phase being highly active from a biological point of view was further supported by the high bone remodeling activity of sclerostin and Wnt inhibitory factor-1 (Wif-1), mediators of the bone anabolic Wnt/β-catenin pathway, two years after Charcot presentation [10].

A limitation of the current study is the small sample size. We believe that the use of a high-sensitivity platform for analysis of biomarkers compensated this limitation by showing that the number of patients in the study was sufficient to achieve a power of 0.8 in an ANOVA comparison of three groups. The current protocol was further strengthened by repetitive measurements of biomarkers yielding a trajectory in support of the individual time points as well as by the well-defined groups of the study.

## Conclusions

In conclusion, proinflammatory cytokines play a critical role in the early and late phase of bone fracture repair. Our results showing that proinflammatory cytokines were not above the level of diabetic controls at Charcot presentation, when patients exerted full weight-bearing on the diseased foot, and increased significantly after offloading, support TCC as a key factor setting off the secondary cascade of inflammatory cytokines responsible for the coupled bone remodeling phase. High IL-1RA/IL-1β ratio in the chronic phase of Charcot is probably a protective measure to reduce further tissue damage induced by renewed full weight-bearing on a deformed foot with impaired bone quality. Additional work is needed to more clearly define the complex cross-talk between pathways involved in the healing of the Charcot foot.
